# Relationship Between Eco Transformational Leadership, Eco Training, and Employee Eco Behavior on Sustainable Corporate Performance of SMEs

**DOI:** 10.3389/fpsyg.2022.900787

**Published:** 2022-05-16

**Authors:** Dina Novita, A. Nururrochman Hidayatulloh, Joseph M. J. Renwarin, Rukun Santoso, Rahayu Mardikaningsih

**Affiliations:** ^1^Fakultas Ekonomi and Bisnis, Universitas Muhammadiyah Surabaya, Surabaya, Indonesia; ^2^Balai Besar Penelitian dan Pengembangan Pelayanan Kesejahteraan Sosial, Yogyakarta, Indonesia; ^3^Institut Teknologi dan Bisnis Kalbis, Jakarta, Indonesia; ^4^Universitas Islam Jakarta, Jakarta, Indonesia; ^5^Universitas Sunan Giri Surabaya, Surabaya, Indonesia

**Keywords:** transformational leadership, eco leadership, employee behavior, sustainable, corporate governance

## Introduction

In the era of digital and industry 4.0 revolution the impact of business activities that can cause damage to the environment results in increased pressure for companies, so companies are required to make eco initiatives as part of their company's CSR programs (Fahlevi and Alharbi, 2021), the Indonesian government requires companies to participate in providing protection for the natural environment and make it part of the company's output. Environmental protection and environmental performance of sustainable companies can be measured by a number of activities carried out by companies in the fields of waste management, pollution control systems, recycling, and mitigation of environmental releases. According to Ayandibu (2019) and Begum et al. (2022) leadership is one of the most important management functions to achieve organizational goals, without a leader the organization will be less efficient and even unable to achieve the vision, mission, and goals specified. The success and continuity of the organization depends on the strength of the leadership because the leader is in charge of controlling the direction to be taken by an organization. So it is concluded that Eco Transformational Leadership is needed to create Employee Eco Behavior which will ultimately make the company's performance run well.

The total number of MSME units in Indonesia currently has reached around 62.9 million units spread across various sectors (Simpen et al., 2019). Around 99.9% of businesses in Indonesia are MSMEs. Apart from GDP and business units, the investment value of MSMEs from 1999 to 2013 also increased rapidly, by 963% to be exact. As of 2018, MSMEs contributed 58.18% of total investment.

According to Sutia et al. (2020); Çop et al. (2021); Lindawati and Parwoto (2021) in running their business in the future, SMEs must pay attention to the environmental impact by starting a green business, starting to switch to using materials that are more environmentally friendly. According to Zafar et al. (2017); Zhang et al. (2020); Widisatria and Nawangsari (2021) low rates, increased production, gaining access to new markets, creating new products or services, and getting other opportunities. By running a green business, it is expected to further advance Indonesian MSMEs by running productive, quality, and environmentally friendly businesses.

Creativity and innovation are the keys to the sustainability of MSMEs during the pandemic in addition to digital adaptation. Creativity and innovation can connect MSMEs to global markets. Currently, MSME actors already know and understand market competition very well, so making unique products has become a must. To make unique products, MSME actors are required to be able to think constructively and creatively, create new innovations so that SME products can develop quickly, and become the key to success in business activities during the pandemic and beyond (Fahlevi et al., 2020) has provided a lot of training and mentoring both online and offline in building creativity and innovation from SMEs.

Leadership is a process of mobilizing a person's ability to influence, move, direct, others by using available resources efficiently and effectively. In SMEs, the leader is one of the key to business success. It's because SME owners play an important role in implementation of business strategies that affect overall organizational performance. So that, It is important for SMEs to know their leadership style appropriate to the characteristics of their business. In accordance with the existing facts and previous research, This study aims to examine the effect of leadership adopted by SME leaders toward employee turnover.

The Effect of Eco Transformational Leadership on Sustainable Corporate Performance. According to Li et al. (2021) in creating sustainable employee and company performance requires environmentally specific transformational leadership. According to Mansoor et al. (2021) and Purwanto et al. (2021) eco transformational leadership is positively related to the suitability of employees' perceived values and significantly affects employees' environmentally friendly behavior. Based on the description above, the hypothesis can be formulated:

H1: Eco Transformational Leadership has a positive and significant effect on Sustainable Corporate Performance.

### The Influence of Employee Eco Behavior on Sustainable Corporate Performance

According to Sun et al. (2022) and Sumarsi (2022) it is said that there is a positive and direct relationship between EGB and organizational sustainability related to environmental sustainability. Where if the employee having eco behavior tends to support the company in achieving performance sustainable company. Based on the description above, the hypothesis can be formulated:

H2: Employee Eco Behavior has a positive and significant effect on Sustainable Corporate Performance

### The Effect of Eco Training on Sustainable on Corporate Performance

According to Sumarsi (2022) and Suprapti et al. (2022) that eco training is seen as an important environmentally friendly practice to significantly increase eco behaviors such as increase employee awareness and knowledge of environmental issues, build positive attitude, taking a proactive approach to environmental issues. According to Singh et al. (2020) and Srour et al. (2020) it is said that through GHRM, especially Eco Training, normal employees are educated and directed to become eco employees in order to achieve the organization's environmental goals and ultimately contribute to the organization's environmental sustainability. Based on the description above, the hypothesis can be formulated:

H3: Eco Training has a positive and significant effect on Sustainable Corporate Performance.

## Method

This research method is quantitative through an online survey, the respondents in this study were 150 SMEs staff and employees and determined by simple random sampling method. The data analysis of this research uses Structural Equation Modeling (SEM) using SmartPLS software.

**Figure 1:**
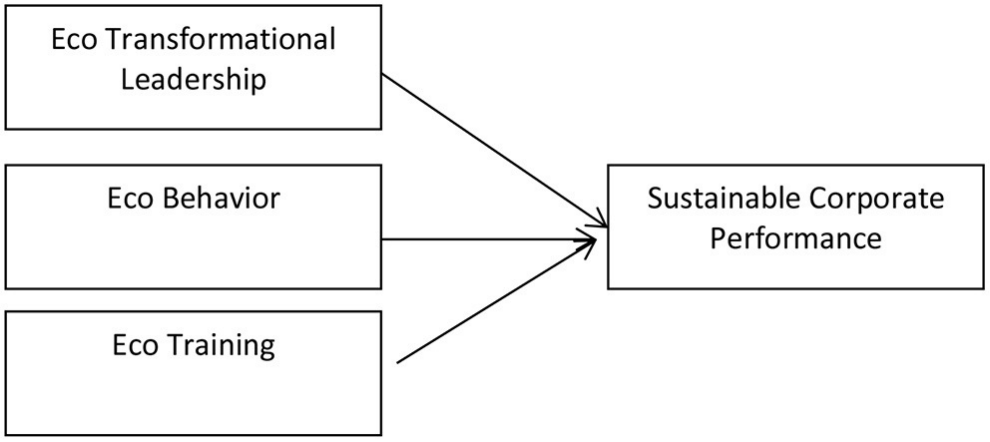
H1: There is a Positive Effect of Eco Transformational Leadership on Sustainable Corporate Performance, H2. There is a Positive influence of Employee Eco Behavior on Sustainable Corporate Performance, and H3. There is a Positive Effect of Eco Training on Sustainable Corporate Performance. Source: Figure is statistic tools generated by authors.

The hypothesis in this study is shown in Figure 1.

## Results and Discussion

H1: There is a Positive Effect of Eco Transformational Leadership on Sustainable Corporate Performance.

Based on the data analysis, the *p*-value is 0.000 <0.050 so it can be concluded that there is a positive effect of Eco Transformational Leadership on Sustainable Corporate Performance. Apart from that it also raises the subordinates' concerns about environmental issues by building good relations with them and then increase the eco values of its subordinates. According to Singh et al. (2020) and Srour et al. (2020) environmental-specific transformational leaders use four dimensions of transformational behavior, namely Eco idealized influence which means acting as a role model for the environment (idealized influence), Eco inspirational motivation which means inspiring followers to engage in environmentally responsible behavior (inspirational motivation), Eco intellectual simulation which means encouraging employees to think about environmental problems in a different way new and creative ideas, Eco individualized consideration which means building good relationships closely with employees to influence their environmental performance (individual considerations).

H2. There is a Positive influence of Employee Eco Behavior on Sustainable Corporate

### Performance

Based on the data analysis, the *p*-value is 0.001 < 0.050 so it can be concluded that there is a positive influence of employee eco behavior on sustainable corporate Performance. The factors that influence Employee Eco Behavior are knowledge, attitudes, motivation and perceived effectiveness, where knowledge and attitudes are important because of their potential impact on behavior formation (Vicente-Molina et al., 2013), Social norms or ethics, work climate and lastly are the values and norms behavior. According to Chen and Chang (2013) and Fernandes and Machado (2021) developed five broad functional categories of Employee Eco Behavior which include: (1) working sustainably, (2) avoiding danger, (3) preserving the environment, (4) influencing others, (5) taking initiative.

H3. There is a Positive Effect of Eco Training on Sustainable Corporate Performance

Based on the data analysis, the *p*-value is 0.001 < 0.050, so it can be concluded that there is a positive effect of Eco Training on Sustainable Corporate Performance. According to Ayandibu (2019) and Begum et al. (2022) creating sustainable company performance requires Eco Human Resources Management (GHRM), in order to increase employee awareness for better quality and commitment to environmental sustainability. One of the important functions of GHRM is Eco Training. According to Çop et al. (2021) stated that the practice of Eco Training is training in order to increase the effect on employees' pro-environmental behavior, when their companies pay more attention to creating a eco climate in the workplace and employees have the opportunity to be trained in the required knowledge and skills and to do eco activities. Eco training and development is training that educate and train employees to master energy-saving work methods, reduce waste, use environmental awareness in organizations, and provide opportunities to involve employees in solving environmental problems.

## Conclusion

Eco Transformational Leadership and Eco Training variables affect the Sustainable Corporate Performance of an organization. The conclusion of this article can be stated in more detail that Eco Transformational Leadership has a positive and significant effect on Sustainable Corporate Performance; Employee Eco Behavior has a positive and significant impact on Sustainable Corporate Performance; Eco Training has a positive and significant effect on Sustainable Corporate Performance. For further research, it is necessary to analyze other variables not examined in this study and use other methods and be carried out in other areas. The government is expected to increase the frequency free training and certification for micro-enterprises so that they can continue their business into a small business or medium. Meanwhile, SMEs are expected can provide guidance to SMEs in a sustainable manner in order to be able to go through certification programs from the government such as ISO, SNI, and so on. The first step to becoming a transformational leader is to build motivation and employee confidence by applying on the job training. Formal training is often difficult to applied to small-scale companies because it can so there is a difference in theory and practice, whereas On the on the job training system, employees can see directly what activities are carried out by leaders so that they can directly learn and practiced. In addition, by holding regular meetings with all employees to exchange ideas and providing solutions to problems that occur in SMEs so that employees feel involved in SME operations. Providing alternative solutions to problems that experienced by employees can open the minds of employees and enhance brand creativity.

## Author Contributions

DN contributed to conceptualization, methodology, investigation, curation, analysis, funding acquisition, and writing. AH and JR helped in investigation, curation, analysis, and writing. RS and RM helped in review, analysis, and writing. All authors contributed to the article and approved the submitted version.

## Conflict of Interest

The authors declare that the research was conducted in the absence of any commercial or financial relationships that could be construed as a potential conflict of interest.

## Publisher's Note

All claims expressed in this article are solely those of the authors and do not necessarily represent those of their affiliated organizations, or those of the publisher, the editors and the reviewers. Any product that may be evaluated in this article, or claim that may be made by its manufacturer, is not guaranteed or endorsed by the publisher.
